# Machine learning methods to predict presence of residual cancer following hysterectomy

**DOI:** 10.1038/s41598-022-06585-x

**Published:** 2022-02-17

**Authors:** Reetam Ganguli, Jordan Franklin, Xiaotian Yu, Alice Lin, Daithi S. Heffernan

**Affiliations:** 1 Brown University, Providence, USA; 2 Department of Computer Sciences, Georgia Institute of Technology, Atlanta, USA; 3 Department of Mathematics, University of Virginia, Charlottesville, USA; 4 Warren Alpert Medical School, Providence, USA; 5 Department of Surgery, Rhode Island Hospital, Brown University, Providence, USA; 6 Division of Trauma/Surgical Critical Care, Division of Surgical Research, Department of Surgery, Rhode Island Hospital, Brown University, Room 207, Aldrich Building, 593 Eddy Street, Providence, RI 02903 USA

**Keywords:** Computational models, Machine learning, Computational biology and bioinformatics, Medical research

## Abstract

Surgical management for gynecologic malignancies often involves hysterectomy, often constituting the most common gynecologic surgery worldwide. Despite maximal surgical and medical care, gynecologic malignancies have a high rate of recurrence following surgery. Current machine learning models use advanced pathology data that is often inaccessible within low-resource settings and are specific to singular cancer types. There is currently a need for machine learning models to predict non-clinically evident residual disease using only clinically available health data. Here we developed and tested multiple machine learning models to assess the risk of residual disease post-hysterectomy based on clinical and operative parameters. Data from 3656 hysterectomy patients from the NSQIP dataset over 14 years were used to develop models with a training set of 2925 patients and a validation set of 731 patients. Our models revealed the top postoperative predictors of residual disease were the initial presence of gross abdominal disease on the diaphragm, disease located on the bowel mesentery, located on the bowel serosa, and disease located within the adjacent pelvis prior to resection. There were no statistically significant differences in performances of the top three models. Extreme gradient Boosting, Random Forest, and Logistic Regression models had comparable AUC ROC (0.90) and accuracy metrics (87–88%). Using these models, physicians can identify gynecologic cancer patients post-hysterectomy that may benefit from additional treatment. For patients at high risk for disease recurrence despite adequate surgical intervention, machine learning models may lay the basis for potential prospective trials with prophylactic/adjuvant therapy for non-clinically evident residual disease, particularly in under-resourced settings.

## Introduction

Gynecologic malignancies account for approximately 12% of all new cancer cases and 15% of all female cancer survivors^1^. Gynecologic malignancies consist primarily of five different anatomic locations: cervical, ovarian, uterine, vaginal, and vulvar cancer^2^. Cervical, uterine, and ovarian cancers accounted for 5.0%, 5.9%, and 2.8% of all worldwide malignancies among women in 2012 respectively^3^. In the United States, approximately 84,000 new cases of gynecologic malignancies are diagnosed resulting in about 2800 deaths annually^4^. Standard management often consists of surgery (i.e., debulking surgery, hysterectomy, and bilateral salpingo-oophorectomy) with neoadjuvant chemotherapy^4,5^. Hysterectomy is the most common surgical procedure in gynecology worldwide^6^. Despite surgery, gynecologic patients often have residual disease, defined as remaining cancer cells after treatment^7–9^.

The presence of this residual disease can often be due to the local inflammation and trauma from the surgical procedure, which causes residual cancer cells to shed into circulation and lead to accelerated micrometastatic growth^10,11^. This was observed as early as the end of the twentieth century, when studies found that cancer patients treated with resection had lower survival rates than cancer patients managed expectantly^12,13^. Given how commonly surgeries are performed for gynecologic cancer patients, patients with gynecologic cancer are at risk of adverse outcomes from surgery, especially with regards to residual disease^14–17^. Residual disease in gynecologic cancer survivors is common, requiring timely intervention to improve survival outcomes^18–21^.

Healthcare providers currently predict risk of residual disease for patients using clinicopathologic and molecular prognostic factors^22–27^. However, identifying individuals at risk for residual disease in the status quo is difficult and the prognosis for recurrent gynecologic malignancy is poor^28–31^. Developing better, more clinically applicable predictive models for risk for residual disease could improve patient outcomes, mainly through identifying patients who could benefit from early intervention and potentially adjuvant therapy^32–35^. Existing prognostic aids are specimen and procedure based and often are specific to a particular type of malignancy^22,36–39^. Furthermore, existing prognostic aids, such as diagnostic radiology may be less accessible in low resource settings^40,41^. As such, there is a need for an automated, machine learning approach to be used alongside conventional clinical data following surgery.

Machine learning (ML) is a field of artificial intelligence in which algorithms develop associations based on existing data to develop statistical models with predictive power over a given dependent variable. Machine learning model development begins with preprocessing data to handle blanks (or NULL values) and organize data numerically in a way that models can accept. This is followed by splitting a given dataset into a “training” set, to which statistical equations are fit in order to develop the predictive model, and “testing” sets, where the developed model’s predictions of the outcome variable are compared against the true values in the “testing” dataset. Machine learning models have begun to show considerable promise in healthcare^42–44^, including models on the American College of Surgeons National Surgical Quality Improvement Program (ACS NSQIP), machine learning models to predict mortality among other end-points, and models aimed at predicting residual malignancy following cytoreduction^38,45–47^. However, there is a lack of studies that have developed machine learning models to predict the presence of residual cancer using health data for post operative hysterectomy patients.

We, therefore, aimed to develop and validate a multivariate machine learning model to predict a given patient’s risk of having postoperative residual malignancy following hysterectomy using easily accessible clinical and laboratory parameters.

## Results

### Patient characteristics

**Figure 1:**
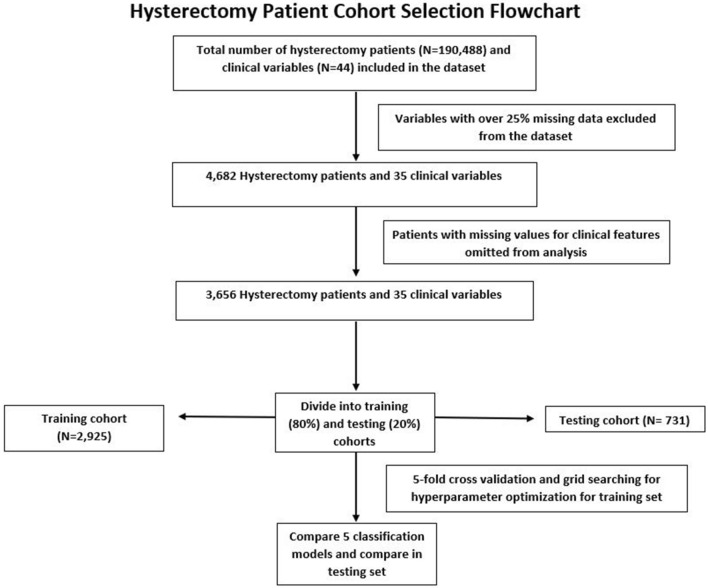
Flow chart showing the hysterectomy patient cohort selection, model training, and performance evaluation processes. A total of 3656 patients were used for model development and were randomly divided into an 80% training set (2925 patients) and a 20% testing set (731 patients). k-fold cross-validation and grid searching for hyperparameters was conducted in the training set, and model performance was evaluated based on area under receiver operating characteristic curves and accuracy rates.

A total of 3656 patients who underwent a hysterectomy for malignancy were extracted from the ACS NSQIP procedure-targeted database over the 14-year period of 2005–2019. For the purposes of this study, the training cohort consisted of 2925 patients (constituting 80% of the dataset) and the testing cohort consisted of 731 patients (20% of the dataset). A flowchart of the patient selection process based on our inclusion criteria is included in Fig. 1.

### Study population characteristics

Of the 3656 patients analyzed, 684 (19%) of these patients were identified to definitively have residual cancer. Only definite “yes” and “no” classifications were used to use the most accurate and applicable data to develop the model. A summary table with descriptive statistics for the residual disease status for each feature in the cohort was developed (Tables 1, 2, 3).

**Table 1:** Clinical history/surgical information summary table.

Characteristic	No residual cancer, N = 2972	Residual cancer, N = 684	p-value^1^	OR	95% Cl
**Parity**			0.12		
0	896 (30%)	196 (29%)		–	–
1	501 (17%)	101 (15%)		0.91	0.67, 1.22
2	840 (28%)	211 (31%)		1.03	0.80, 1.32
3	436 (15%)	90 (13%)		0.95	0.69, 1.30
4+	299 (10%)	86 (13%)		1.38	0.99, 1.92
**Prior abdominal operations**			0.33		
No	2176 (73%)	514 (75%)		–	–
Yes	796 (27%)	170 (25%)		0.85	0.68, 1.06
**Prior pelvic operations**			0.57		
No	1717 (58%)	404 (59%)		–	–
Yes	1255 (42%)	280 (41%)		0.95	0.78, 1.16
**Endometriosis**			< 0.001		
No	2755 (93%)	666 (97%)		–	–
Yes	217 (7.3%)	18 (2.6%)		0.58	0.33, 0.97
**Endometriosis location**			< 0.001		
No	2755 (93%)	666 (97%)		–	–
Yes	217 (7.3%)	18 (2.6%)			
**Pelvic inflammatory disease**			0.40		
None	2920 (98%)	677 (99%)		–	–
Inflammatory	45 (1.5%)	6 (0.9%)		0.75	0.27, 1.77
Tube-ovarian abscess	7 (0.2%)	1 (0.1%)		1.17	0.06, 7.69
Uterine weight (g)	207 ± 13	186 ± 23	0.11	1.00	1.00, 1.00

*OR* odds ratio, *CI* confidence interval.

^1^Continuous Variable: one-way ANOVA, Binary Variable: Fisher’s Exact Test, Categorical Variable: Chi-Squared Test.

**Table 2:** Cancer related variables summary table.

Characteristic	No residual cancer, N = 2972	Residual cancer, N = 684	p-value^1^	OR	95% Cl
**Size of grossly visible tumor**			0.016		
Less than 1 cm	315 (11%)	97 (14%)		–	–
1–2 cm	442 (15%)	87 (13%)		0.59	0.41, 0.85
Greeter than 2 cm	2215 (75%)	500 (73%)		0.65	0.49, 0.87
**Gross abdominal disease-lymph nodes**			0.12		
No	2256 (76%)	499 (73%)		–	–
Yes	716 (24%)	185 (27%)		1.07	0.86, 1.34
**Gross abdominal disease-bowel serosa**			< 0.001		
No	2207 (74%)	292 (43%)		–	–
Yes	765 (26%)	392 (57%)		1.67	1.36, 2.04
**Gross abdominal disease-rowel mesentery**			< 0.001		
No	1651 (56%)	129 (19%)		–	–
Yes	1321 (44%)	555 (81%)		1.90	1.49, 2.43
**Gross abdominal disease-live**			< 0.001		
No	2817 (95%)	562 (82%)		–	–
Yes	155 (5.2%)	122 (18%)		1.56	1.15, 2.10
**Gross abdominal disease-spleen**			< 0.001		
No	2886 (97%)	619 (90%)		–	–
Yes	86 (12.9%)	65 (9.5%)		1.04	0.71, 1.52
**Gross abdominal disease-diaphragm**			< 0.001		
No	2651 (89%)	360 (53%)		–	–
Yes	321 (11%)	324 (47%)		3.57	2.88, 4.44
**Gross abdominal disease-pelvis**			< 0.001		
No	1410 (47%)	152 (22%)		–	–
Yes	1562 (53%)	532 (78%)		1.81	1.46, 2.26
**Cervical cancer FIGO stage**			< 0.001		
0–1B2	164 (5.5%)	3 (0.4%)		–	–
II–IVB	100 (3.4%)	16 (2.3%)		3.07	0.91, 14.1
Not a cervical cancer case	2708 (91%)	665 (97%)		3.86	1.37, 16.2
**Corpus uteri cancer stage**			< 0.001		
0–II	561 (19%)	19 (2.8%)		–	–
IIA–IIIC	580 (20%)	142 (21%)		3.88	2.33, 6.81
IV–IVB	29 (1.0%)	14 (2.0%)		6.19	2.60, 14.5
Not a corpus uteri cancer case	1802 (61%)	509 (74%)		2.93	1.74, 5.18
**Ovarian cancer stage**			< 0.001		
I–III	560 (19%)	42 (6.1%)		–	–
IIIA–IV	1050 (35%)	457 (67%)		2.23	1.56, 3.24
Not an ovarian cancer case	1362 (46%)	185 (27%)		1.68	1.09, 2.62

*OR* odds ratio, *CI* confidence interval.

^1^Continvous Variable: one-way ANOVA, Binary Variable: Fisher’s Exact Test, Categorical Variable: Chi-Squared Test.

**Table 3:** Clinical complication outcome variables summary table.

Characteristic	No residual cancer, N = 2972	Residual cancer, N = 684	p-value^1^	OR	95% CI
**Intestinal obstruction**			0.007		
No	2874 (97%)	646 (94%)		–	–
Yes	98 (3.3%)	38 (5.6%)		0.83	0.50, 1.36
**Prolonged postoperative NPO or NGT use**			< 0.001		
No	2769 (93%)	576 (84%)		–	–
Yes	203 (6.8%)	108 (16%)		1.79	1.29, 2.49
**Anastomotic leak**			0.68		
No	2941 (99%)	675 (99%)		–	–
Yes	31 (1.0%)	9 (1.3%)		0.47	0.18, 1.13
**Ureteral obstruction**			0.96		
No	2962 (100%)	681 (100%)		–	–
Yes	10 (0.3%)	3 (0.4%)		0.61	0.12, 2.45
**Ureteral fistula**			> 0.99		
No	2970 (100%)	683 (100%)		–	–
Yes	2 (< 0.1%)	1 (0.1%)		3.21	0.12, 52.7
**Bladder fistula**			0.52		
No	2969 (100%)	682 (100%)		–	–
Yes	3 (0.1%)	2 (0.3%)		3.30	0.40, 23.4

*OR* odds ratio, *CI* confidence interval.

^1^Continuous Variable: one-way ANOVA, Binary Variable: Fisher’s Exact Test, Categorical Variable: Chi-Squared Test.

### Model variable importance

**Figure 2:**
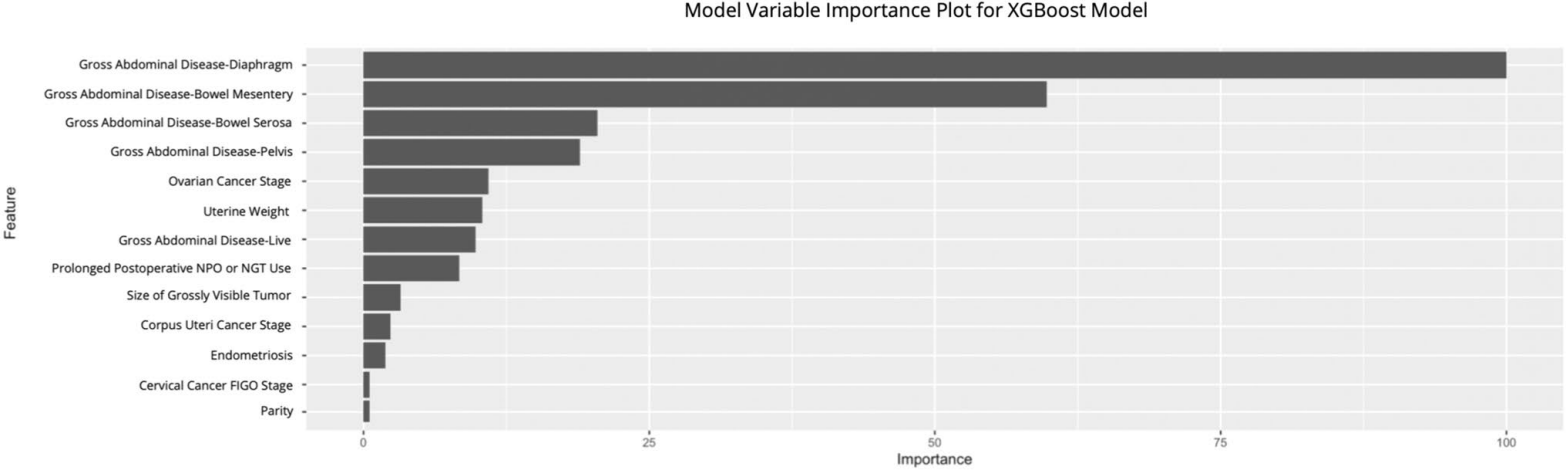
Analysis of the importance of each variable in the XGBoost machine learning model. The histogram describes the relative importance of all 35 clinical features in the logistic regression model. The relative importance is quantified by assigning a weight between 0 and 100 for each variable.

5 machine learning models based on Random Forest, eXtreme Gradient Boosting (XGBoost), Logistic Regression (LR), Support Vector Machine (SVM), and K-Nearest Neighbor (KNN) algorithms were created. The logistic regression, random forest, and XGBoost models were the 3 highest performing models. 35 statistically significant clinical parameters were included within these models. The algorithm and methodology we used to obtain our model variable importance plots have been previously cited in the literature^48–50^. The top postoperative predictors of residual disease factored across the top three models were the presence of malignancy located on the diaphragm, disease located on the bowel mesentery, disease located on the bowel serosa, and disease located within the adjacent pelvis prior to surgical debridement. Specifically, within the XGBoost model, the top post-operative predictors of residual disease were the presence of malignancy located on the diaphragm, disease located on the bowel mesentery, and disease located on the bowel serosa (Fig. 2). A more comprehensive chart of ranked variable importances can be found in Supplemental Figs. S1–S3 for the full XGBoost, logistic regression, and random forest models. Supplemental Figure S4 has the variables ranked as having little or no importance to the XGBoost model. The variables with the largest odds ratios were presence of a bladder fistula (OR 3.05), presence of a urethral fistula (OR 3.04), low cervical cancer staging, and presence of gross abdominal disease in the Diaphragm (OR 3.37).

### Model performance

The Extreme Gradient Boosting model had an AUC of 0.90 (95% CI 0.87–0.93), with an accuracy of 87.3%. The Random Forest model had an AUC of 0.90 (95% CI 0.87–0.93), with an accuracy rate of 87.3%. The Logistic Regression model had an AUC of 0.90 (95% CI 0.87–0.93) with an accuracy rate of 87.0%. The K-nearest-neighbors model had an AUC of 0.70 (95% CI 0.65–0.76), with an accuracy of 80.8%. The support vector machine model had an AUC of 0.59 (95% CI 0.53–0.65), with an accuracy of 80.4% (Table 4).

**Table 4:** Comparative chart displaying the accuracy score, area under the receiver operating characteristic curve, F1 score, and Matthews Correlation Coefficient (MCC) for each individual machine learning model.

Model	Accuracy	ROC AUC	F1	MCC
Logistic regression	88% (85–90%) p < 0.05	0.89 (0.86–0.92) p < 0.05	0.74 (0.69–0.78) p < 0.05	0.50 (0.42–0.58) p < 0.05
Random forest	88% (86–90%) p < 0.05	0.88 (0.84–0.91) p < 0.05	0.72 (0.67–0.77) p < 0.05	0.52 (0.44–0.60) p < 0.05
XGBoost	87% (85–90%) p < 0.05	0.88 (0.85–0.91) p < 0.05	0.73 (0.69–0.78) p < 0.05	0.51 (0.42–0.59) p < 0.05
K-nearest neighbors	81% (80–86%) p < 0.05	0.72 (0.67–0.76) p < 0.05	0.53 (0.49–0.58) p < 0.05	0.19 (0.09–0.28) p < 0.05
Support vector machine	81% (80–85%) p < 0.05	0.50 (0.44–0.56) p < 0.05	0.45 (0.44–0.46) p < 0.05	0.0 (0.00–0.00) p < 0.05

A 95% confidence interval is listed for each metric in parentheses, and a p-value expressing if a metric for each model is significantly different from the same metric from all the other models is listed.

**Figure 3:**
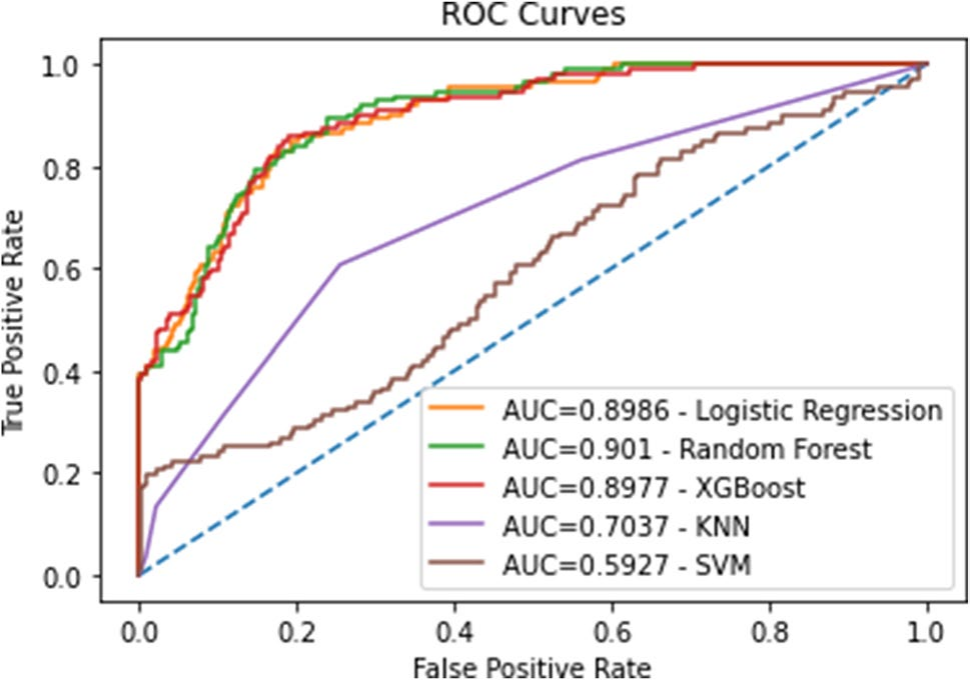
Evaluation of the machine learning models’ predictive abilities: receiver operating characteristic curves of all models plotted, along with area under the curve for each model listed in the legend.

The XGBoost, Random Forest, and Logistic Regression models all had comparable AUC and accuracy metrics, outperforming the SVM and KNN models (Fig. 3). The accuracy rates of these top 3 models outperform the current rate of residual disease diagnosis by healthcare providers.

## Methods

Data was extracted from the ACS NSQIP procedure-targeted database from the time period of January 2005 to December 2019. Patients who underwent a hysterectomy for a known malignancy were included within the extracted dataset. The ACS NSQIP database is a national surgical registry used to track risk-adjusted outcomes after surgical procedures from any medical specialty. Prospective variables are obtained and audited by trained clinical reviewers. The American College of Surgeons National Surgical Quality Improvement Program and the hospitals participating in the ACS NSQIP are the source of the data used herein; they have not verified and are not responsible for the statistical validity of the data analysis or the conclusions derived by the authors.

The inclusion criteria for this experiment were: all hysterectomy patients in the ACS NSQIP procedure targeted database, collected between the 2005 and 2019 calendar years, without missing values for any clinical features, and with over 75% of their clinical data present. The exclusion criteria were: any non-hysterectomy patient and any hysterectomy patient with over 25% missing values; hysterectomy patients who had any missing values for their clinical features were also excluded.

### Outcome

The primary outcome was the presence of gross residual disease following a hysterectomy procedure for malignancy. Within the ACS NSQIP dataset, this variable is either coded as a “No”, “Yes”, or coded as “NULL” in cases where it was either not recorded, or not possible to identify. The presence of gross residual disease was defined as *any portion of the metastatic tumor which remains after surgical procedure, by the ACS NSQIP clinical support team.*

Patients carrying blank/NULL values for the primary outcome variable column (Gross residual disease) were removed, during preprocessing to eliminate any uncertainty/inaccuracy from the training.

The study’s primary aim was to construct comparable models with improved parameters, which would yield a risk predictor for residual cancer after a hysterectomy procedure. Each predictor (clinical and laboratory variables) was studied for their odds ratios within a 95% confidence interval (CI).

### Machine learning models

The entire cohort of hysterectomy patients were converted into numeric variables. Continuous numeric variables were left as is, and binary “yes” or “no” responses were changed to 1’s and 0’s respectively.

The initial development cohort was 190,488 patients with 44 clinical variables. Any patient with missing data pertaining to the presence or absence of gross residual disease were excluded. Columns with over 25% of the data missing were also dropped to reduce inaccuracy. No imputations were used in the development or validation cohort for the dataset to reduce erroneous bias. This left 4682 patients for further analysis.

Multicollinearity was assessed by creating a heatmap correlation matrix to omit variables with high variance inflation factors (VIFs) to preserve the integrity of the statistical significance of the input variables for the model. Any variables with a VIF greater than 3 were dropped from the model. Variables were dropped one by one, starting with the variables with the highest VIF, after which the list of VIFs for all features included in the model were reevaluated to see if they were all under or over 3. If all the variables included in the model did not have a VIF under 3, then the next variable with the highest VIF was dropped from the model.

This left 3656 patients for the final analysis. Grid search was performed on the dataset, which was split into an 80–20% train-test split, where 80% of the data was used to train a logistic regression model, random forest, and extreme gradient boosting model. Within the 80% training set, the outcome variable of gross residual disease was dropped from the data frame prior to the training split process to avoid skewing predictive potential.

The other 20% was used to test the individual accuracy of each model, respectively. This was done to search for the estimator with the optimal hyperparameter values. During the search, fivefold cross validation was performed on estimators of different hyperparameter values, and the estimator with the largest mean cross-validated score was selected as the optimal model from the grid search. Hyperparameters are properties of a model that can be tweaked to control its learning process, at the cost of lengthening execution time, should too many be added. In the past decade, studies have shown methods that were developed to rank hyperparameters by importance, typically by how much is gained for a metric such as accuracy or AUC, based on multiple datasets. For each classification model, hyperparameters were chosen which were highly ranked for that model across multiple datasets as indicated in the literature^51,52^.

The cohort was split at the patient level such that no training data could appear in the testing set. All variables were included in the model to optimize the predictive potential without introducing background noise.

To mitigate bias, the data was checked for any high multicollinearity (intercorrelation between any two variables) to see if there are any features to consider removing that could negatively impact the model’s prediction accuracy. Features with high multicollinearity were omitted to minimize bias within the model’s predictions. We generated a correlation heatmap and also computed the variance inflation factor for each variable to screen for any features which may have introduced bias, and nothing indicated high multicollinearity. We also performed cross validation on our models, which reduced bias and variance to prevent the models from overfitting onto the data.

### Statistical analysis

Descriptive statistical analysis was conducted on the data based on the patient’s presence of gross residual disease, as recorded in the NSQIP. Initial analysis was done by conducting an independent, one-way analysis of variance (ANOVA) test of every continuous, numeric variable included in the model, a chi-squared test for every categorical variable in the model, and Fisher exact test for binary variables, partitioned between patients who did and did not have a diagnosis of gross residual disease.

The ML models were constructed from the training cohort and assessed on the validation cohort, independent from model development, by calculating the area under the curve (AUC) of the model’s receiver operating characteristic (ROC). The AUC, plotting the odds of a false positive against the odds of a true positive, was used due to its threshold independent nature to describe the model’s classification ability. A 95% CI for the models’ AUC was obtained through bootstrapping.

All analyses were conducted using the Sklearn version 0.24.1 (https://scikit-learn.org/stable/about.html) package^53^ in Python (Python Software Foundation)^54^ and R 4.1.0 (https://www.R-project.org/)^55^. Python version 3.8.8 (https://www.python.org/downloads/release/python-388/) was used for analysis.

## Discussion

This machine learning cohort study demonstrated the feasibility of applying machine learning models on a large, heterogeneous population of hysterectomy patients in order to forecast the presence of gross residual disease postoperatively.

In the setting of tumor excision surgeries, there exist 2 possibilities: cases where surgeons definitively have been able to identify or rule out the postoperative presence of gross residual disease via visual inspection and/or pathology scans and cases where there exists a medical uncertainty as to whether there is any remaining disease left in the patient. The latter possibility constitutes a serious clinical problem for physicians, who then must decide how to proceed with postoperative patient management and weigh the risk between preventing possible residual disease with adjuvant chemotherapy, at the cost of harming the patient’s health.

Our dataset was obtained from the ACS NSQIP. Previous studies have described the significance of clinical features in the ACS NSQIP to predict surgical outcomes for gynecologic procedures^56^. Here, we use machine learning models to automate this process. Machine learning models were prioritized over deep learning models in our study due to their faster run time, lesser need for computational power, and easy interpretability (like through the generation of model variable importance plots which can highlight key clinical features pertinent to a given outcome variable) which are all vital for implementation in low resource settings. Deep learning models need more computational power, take longer to run, have lower interpretability, and are better suited for more complex problems/prediction tasks where an organized data frame is not present.

Our machine learning models were trained on definitively diagnosed cases of residual disease versus no residual disease, but can be generalized for cancer patients, particularly those in low resource settings, whose residual disease status is unclear, to give direction to the surgeon for the patient’s postoperative clinical management. This study can serve as the basis for prospective trials with prophylactic chemotherapy for non-clinically evident residual disease.

Predicting residual disease after hysterectomy would improve treatment planning. Given the poor prognosis of recurrent gynecological cancers, there is a strong need for tools to identify gynecologic cancer patients at risk for residual disease following surgical procedures. Patients at high risk could be monitored more closely or moved directly to additional chemotherapy and radiation therapy. Machine learning can be used successfully for disease diagnosis and prediction^57^.

Previous studies have made an attempt to develop models predicting risk of residual disease following surgery. In 2018, Horowitz et al. published a predictive model for microscopic residual disease following complete cytoreduction in patients with advanced epithelial ovarian cancer. While this study identified many variables predictive of residual disease at cytoreduction, the area under the curve of the receiver operating characteristic was 0.73, putting into question the predictive ability of the model^38^. In a more recent study, Kumar et al. reported computed tomography prediction models for residual disease at primary debulking surgery for advanced ovarian cancer. The model predicting gross residual disease had the highest predictive value, with its c-index reaching 0.762^50^.

In our work, we present a machine learning model to establish risk models (LR, KNN, SVM, RF, and XGboost models) that combine clinical and operative parameters to identify patients with increased risk of residual disease following hysterectomy. The top three performing models: XGBoost, Logistic Regression, and Random Forest models all had statistically similar ROC curves and accuracy rates. Our model was trained and validated on 3656 patients and showed consistent calibration across the database. The cohort was representative of hysterectomy patients across the United States^58^. Though we had a different end goal, our model was competitive with results published in literature for other machine learning-based studies^59–61^.

Our study approach had several strengths. Due to the nature of the data collected, such an approach could be applied to other cancers following a surgical procedure as well. In 2012, an estimated 8.2 million cancer deaths and 14.1 million new cancer cases occurred worldwide^62^. Accurately predicting residual disease in different cancers could lead to considerable reductions in healthcare costs while also improving long-term survival for cancer patients. Additionally, a prognostic approach based on clinical and operative parameters would be accessible to low resource settings as well. This analysis could be implemented in other countries that have large healthcare databases, such as Japan, without requiring additional data collection^63^. Furthermore, we included a detailed calibration assessment, which suggests our model would be well calibrated in other databases.

Our proposed approach had important limitations. First, while our model does not impute any values, only definitive positives for residual cancer were counted. Patients for whom the residual cancer status was uncertain could not be used for the development of the model, as surgeons were not able to definitively stage these patients. This means that the model may be biased towards more clearly defined cases where there is gross residual cancer and may not perform as well for patients for whom it is hard to discern the gross residual cancer status. However, with clinical validation, model training on increased sample sizes can hopefully lead to application on clinically ambiguous patients as well. Furthermore, greater consistency and fewer missing input values would improve the model’s discrimination. Second, these machine learning models were trained on the ACS NSQIP database and, despite thorough feature selection and hyperparameter optimization, may be fit for the nuances of the NSQIP data specifically. To overcome this limitation and to increase generalizability, these models should be tested in other oncology settings, with a mixture of diversified data sources to best assess generalizability. Doing so may help capture other significant parameters and, using a richer data source, achieve more competitive performance. Finally, though we can interpret the model’s decisions and variable splitting to identify patients at higher risk, the model only captures correlations in data and not causal pathways.

The only potential safety issue in utilizing AI systems to analyze patient data would theoretically be a breach of patient privacy. To avoid this, all features used to develop any models should be fully deidentified. In our research, we were able to mitigate this by using solely deidentified data to train our models, so no model can attribute given clinical features to the original patient, as that data was never shown to the model. Furthermore, because machine learning models are governed by statistical equations, it is impossible to “reverse engineer” machine learning models to uncover the original patient data, as the models were built on the entire aggregated patient data. To mitigate anyone from potentially trying to use the statistical equations of machine learning models to infer aggregate attributes about the original data, firewalls and secure deployment services can be used to ensure that it is impossible for anyone to be able to view/analyze the models.

Our machine learning models were trained on definitively diagnosed cases where the presence or absence of gross residual disease was known; our models can be extrapolated for the vast majority of non-clinically evident cases of gross residual disease, where there is clinical uncertainty to guide adjuvant therapy and/or postoperative follow-up. This will be most clinically useful at the end of index operations where surgical teams believe they have removed all cancer but have missed residual disease. In these settings, our machine learning models can predict the possibility of residual disease and risk stratify patients to alter their postoperative management. Our research serves as the basis for prospective studies on patients with non-clinically evident remaining cancer who are believed to not have residual disease but have a high risk score on our model.

Our findings suggest that machine learning methods, specifically Logistic Regression, Random Forest, and Extreme Gradient Boosting models, have strong classification ability and hold potential for clinical application to guide patient management, improve patient outcomes, and modulate treatment regimen, particularly for low resource settings with primarily clinical and operative variables available for analysis.

This model can have a dual integration modality depending on the clinical care setting. In developed settings, this model can be deployed publicly as a software as a service cloud platform, which healthcare facilities can directly integrate into their EHRs for dynamic prediction based on the available EHR data. The model would then generate a personalized risk score for the patient’s likelihood of residual disease, prompting healthcare providers to initiate sooner follow-up care and initiate adjuvant therapy. In low-resource settings that lack EHRs but have a prevalence of mobile devices, this model would be a mobile app, where healthcare providers can manually enter in necessary clinical features to receive the risk score output for each patient, indicating further therapy/closer follow-up.

## Conclusion

Existing residual disease prognostic methods are time intensive, require pathology specimens, and often are restricted to modelling only one particular type of cancer. Current prognostic aids require expensive tools and are largely inaccessible in low resource settings. Our findings can streamline clinical postoperative diagnosis and serve as a novel lens to utilize commonly collected operative parameters for the prediction of residual disease using machine learning.

## Supplementary Information

Supplementary Legends.

Supplementary Figure S1.

Supplementary Figure S2.

Supplementary Figure S3.

Supplementary Figure S4.

## Data Availability

The datasets generated during and/or analysed during the current study are available from the corresponding author on reasonable request.
