# Supplementary material for: Machine learning methods to predict presence of residual cancer following hysterectomy
Source: Sci Rep. 2022 Feb 17;12:2738. doi: 10.1038/s41598-022-06585-x (PMC8854708; doi:10.1038/s41598-022-06585-x)
Supplement: Supplementary file 1 — Supplementary Legends. [file 41598_2022_6585_MOESM1_ESM.docx]

Supplemental Figure 1: Full model variable importance plot of logistic regression model

Supplemental Figure 2: Full model variable importance plot of random forest model

Supplemental Figure 3: Full model variable importance plot of XGBoost model

Supplemental Figure 4: Insignificant variables with no importance ranking in XGBoosting model; included to show which clinical features are less important in determination of residual disease
