# Supplementary figures and images for: Machine learning methods to predict presence of residual cancer following hysterectomy
Source: Sci Rep. 2022 Feb 17;12:2738. doi: 10.1038/s41598-022-06585-x (PMC8854708; doi:10.1038/s41598-022-06585-x)

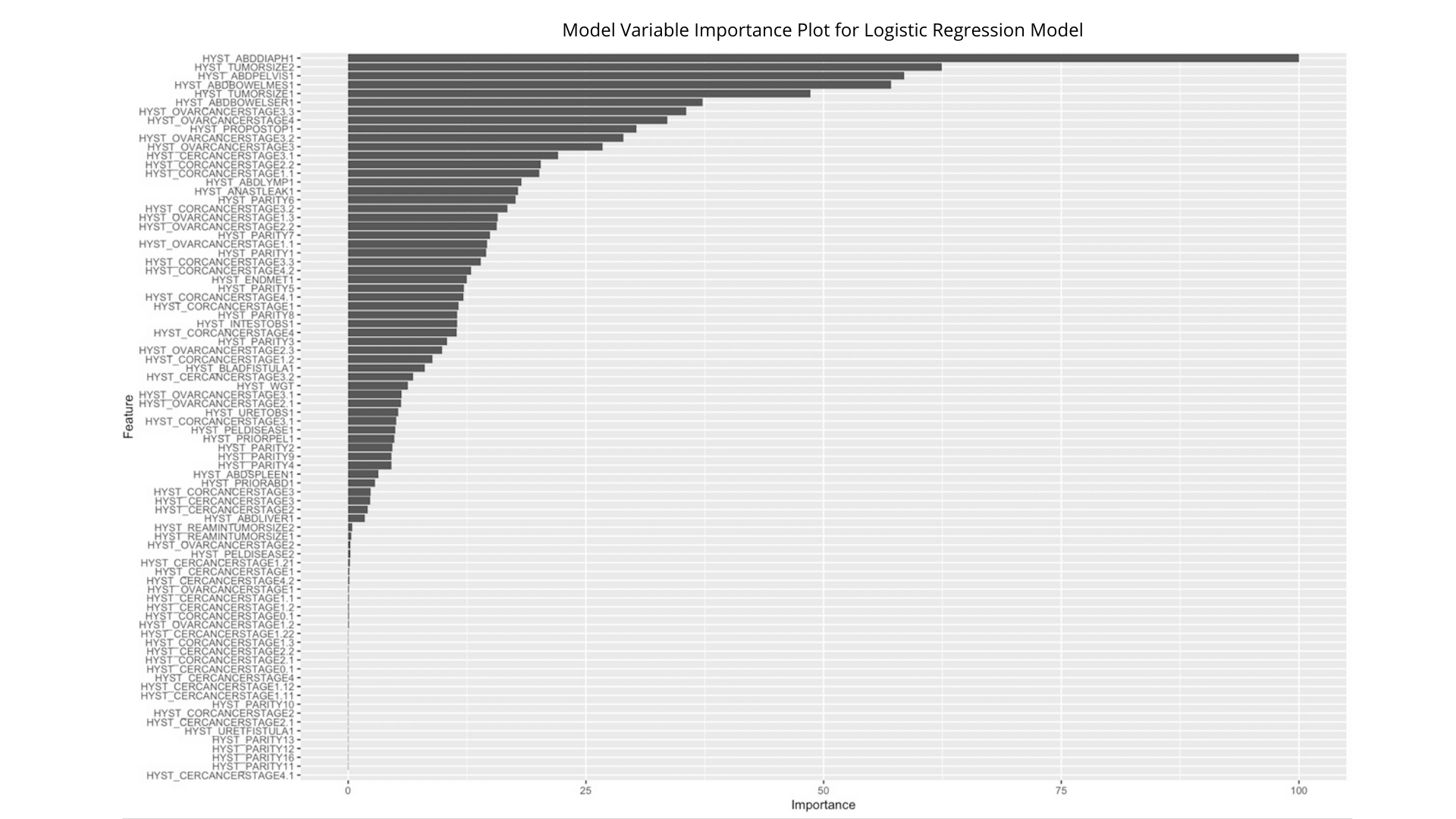

Supplement: Supplementary file 2 — Supplementary Figure S1. [file 41598_2022_6585_MOESM2_ESM.png]

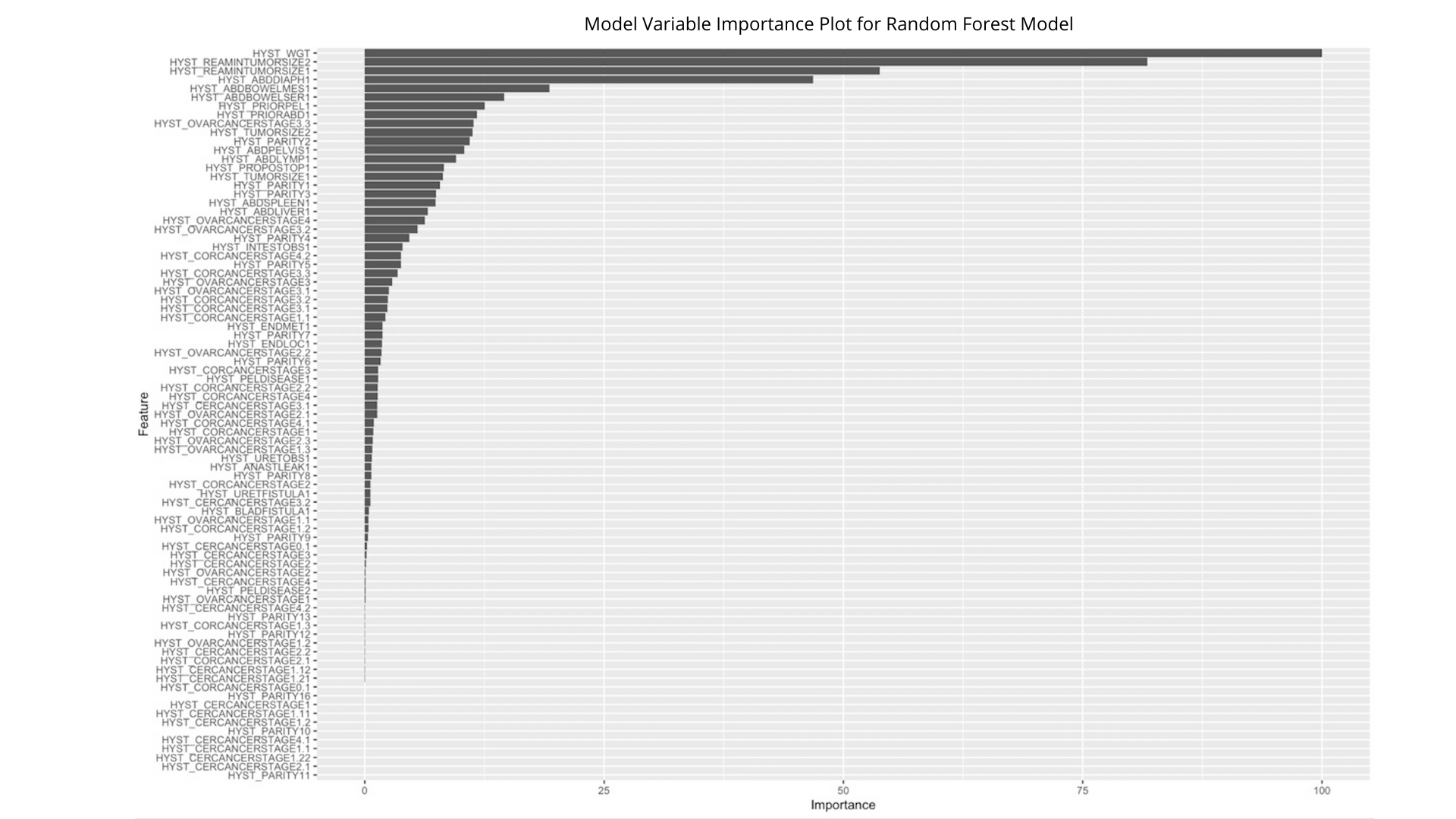

Supplement: Supplementary file 3 — Supplementary Figure S2. [file 41598_2022_6585_MOESM3_ESM.png]

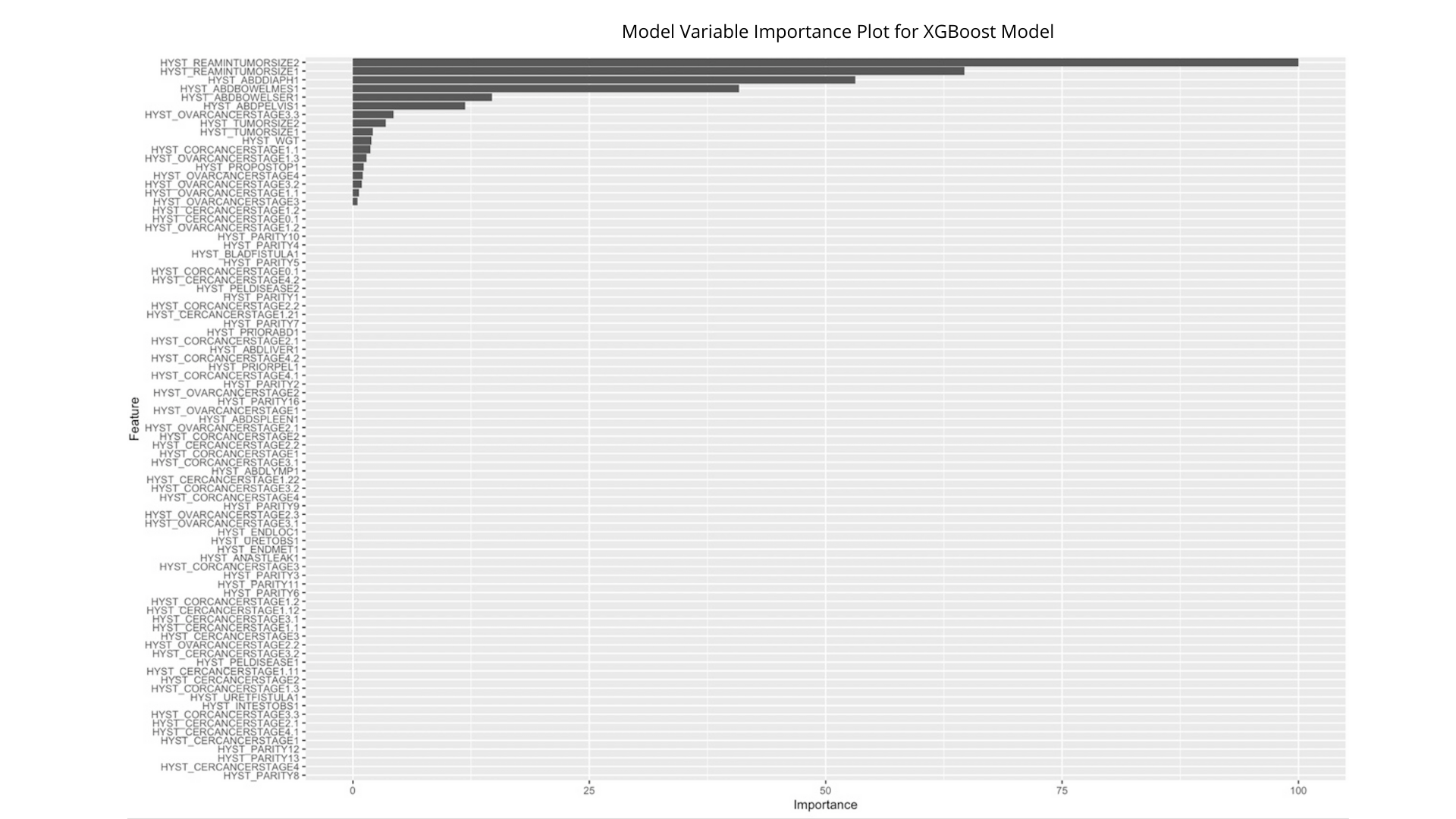

Supplement: Supplementary file 4 — Supplementary Figure S3. [file 41598_2022_6585_MOESM4_ESM.png]

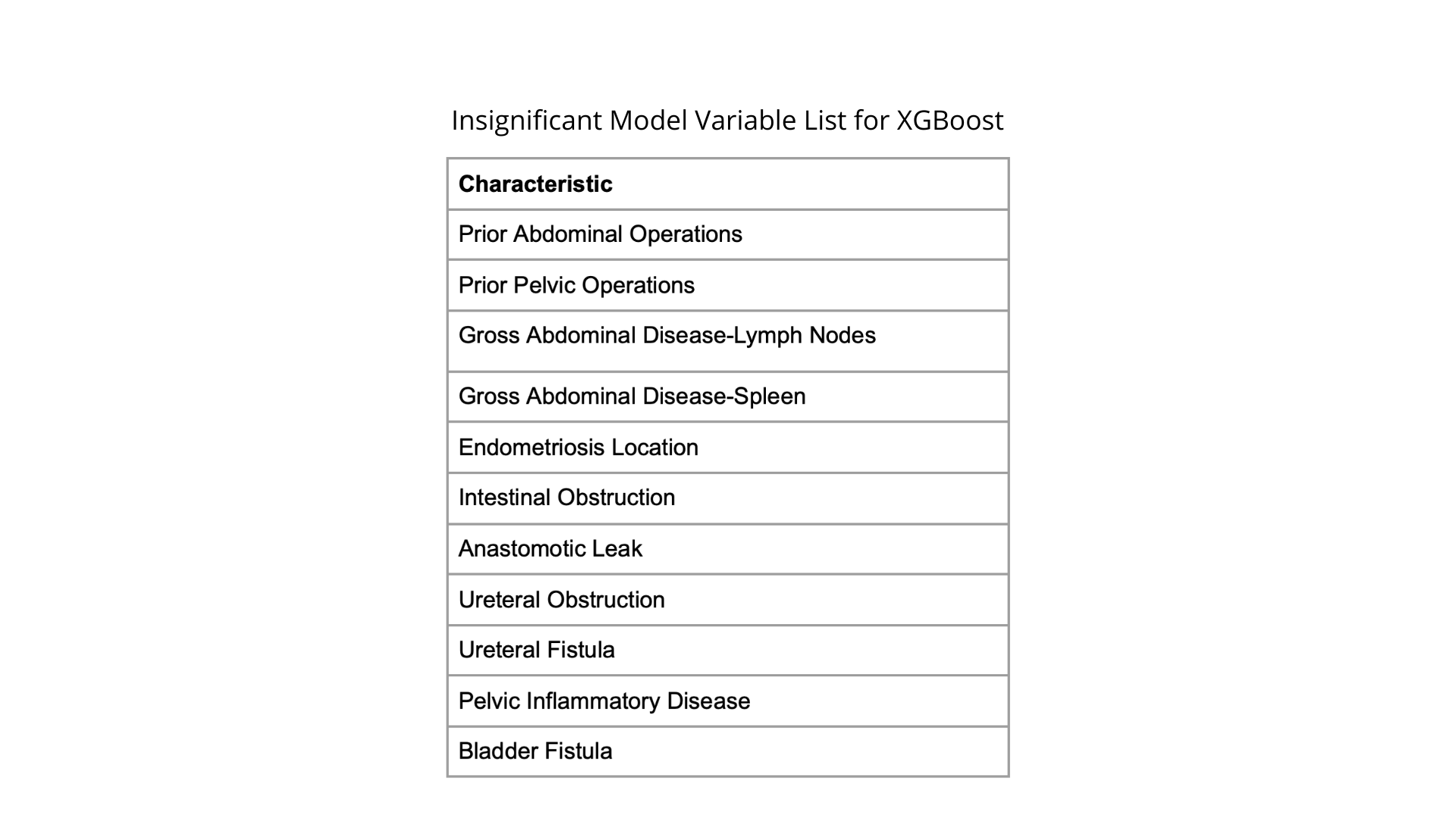

Supplement: Supplementary file 5 — Supplementary Figure S4. [file 41598_2022_6585_MOESM5_ESM.png]
